# Prevalence and Predictors of Carpal Tunnel Syndrome Symptoms Among Teachers in Riyadh: A Cross-Sectional Study

**DOI:** 10.7759/cureus.35040

**Published:** 2023-02-15

**Authors:** Ahmed H AlHussain, Alwaleed A Alshahir, Faisal H AlNaqa, Ehab F Alsaygh, Ibrahim A Alquwaiz, Mohammed S Alqahtani

**Affiliations:** 1 Orthopaedic Surgery, King Abdulaziz Medical City, Riyadh, SAU; 2 College of Medicine, King Saud Bin Abdulaziz University for Health Sciences, Riyadh, SAU; 3 Medical Research, King Abdullah International Medical Research Center, Riyadh, SAU; 4 College of Medicine, Taibah University, Medina, SAU; 5 College of Medicine, Prince Sattam Bin Abdulaziz University, Al-kharj, SAU; 6 College of Medicine, King Khalid University, Abha, SAU

**Keywords:** carpal tunnel syndome, saudi arabia, boston carpal tunnel questionnaire, median neuropathy, teachers, schoolteachers

## Abstract

Background

Carpal tunnel syndrome (CTS) is a musculoskeletal disorder (MSD) afflicting the upper limbs with a prevalence of approximately 14.4% in the general population. Previous studies have noted the increasing prevalence of MSDs among teachers but have not investigated in depth the prevalence and predictors of CTS symptoms in this population. The aim of this study was to help fill this gap in the literature by investigating teachers working in Riyadh, Saudi Arabia.

Methods

We conducted this cross-sectional study in Riyadh using an online survey. We distributed the Boston carpal tunnel questionnaire (BCTQ) to schoolteachers in the city through the social media applications Twitter, WhatsApp, and Telegram. We assessed the respondents’ symptoms using Univariate association analyses with a Wilcoxon rank sum test for the continuous variables and Fisher’s exact test and Pearson’s chi-squared test for the categorical variables. We assessed the independent risk factors for CTS by constructing multivariate binary logistic regression models and expressed the results using the odds ratio (OR) and 95% confidence intervals (95% CIs), with p < 0.05 indicating statistical significance.

Results

The sample for this study included 490 teachers. Among them, the prevalence of moderate to severe CTS symptoms was 40.0%, and self-reported CTS was 9.1%. The teachers who were female, relatively old, left-handed, retired, and spent significant time using a pen, keyboard, and/or blackboard were more likely than those who were male, relatively young, right-handed, and did not spend significant time using a pen, keyboard, and/or blackboard to self-report CTS and exhibit moderate to severe symptoms.

Conclusions

We found a relatively high percentage (40.0%) of CTS symptoms among teachers working in Riyadh. This finding suggests that any sign of CTS symptoms should be checked to ensure early diagnosis and treatment, which contribute to positive outcomes, particularly given the recent increase in such risk factors for CTS as diabetes, hypothyroidism, and high BMI in populations worldwide.

## Introduction

Carpal tunnel syndrome (CTS) is a musculoskeletal disorder (MSD) that afflicts the upper limbs with a prevalence of 14.4% in the general population [1]. The etiologies of the disorder include the work involved in certain occupations [2]. Teaching is among the occupations associated with MSDs, which afflict an estimated 35% to 95% of educators [3]. The numerous ergonomic and work-related factors that predispose this population to CTS include repeated flexion and extension of the wrist and other wrist movements associated with long hours spent grading assignments, preparing lessons, performing nonteaching clerical duties, attending developmental courses, and participating in extracurricular activities with students [4]. Furthermore, in recent years, the increasing rates of metabolic disorders such as diabetes, hyperthyroidism, and obesity have added to the burden on healthcare systems worldwide, and these disorders are considered risk factors for CTS, especially in the context of stressful work [5-7]. Unsurprisingly, the early diagnosis of CTS is associated with more favorable post-surgical outcomes than is the case when treatment begins long after symptoms have started [8]. Therefore, when teachers present with clinical symptoms of CTS, caregivers should take measures to facilitate early diagnosis and, thereby, improve treatment outcomes.

Studies in several countries have reported on the prevalence of MSD in teachers, with reports of wrist/hand pain specifically occurring at a prevalence of 13.4% in Turkey, 26% in Bolivia, 40.7% in Malaysia, and 44% in the Philippines [9-12]. Reports in other studies of the prevalence of wrist pain in Saudi Arabia range from 7.4% to 22.1% [13-16]. However, these studies address wrist/hand pain as a symptom without exploring its characteristics or relationship to CTS in teachers. To our knowledge, only Stevens et al. [17] and Younis et al. [18] have reported the prevalence of CTS among teachers, offering widely divergent estimates of 2.9% and 62%, respectively. In view of the increase in MSDs among teachers and of hand and wrist pain in particular, we reasoned that CTS, a common MSD, would be similarly prevalent among teachers.

Accordingly, the aim of this study was to estimate the prevalence of CTS symptoms and assess the factors associated with it among schoolteachers working in Riyadh, Saudi Arabia.

## Materials and methods

For this cross-sectional study, we distributed an online-based survey through the social media applications Twitter, WhatsApp, and Telegram to the members of teachers’ groups in Riyadh and visited schools to encourage participation. The data of this study were collected during the months of January and February 2022.

Eligibility criteria

Male and female teachers who had been working for at least a year were included in the study. Any teachers with a history of orthopedic trauma or congenital disorders of the wrist were excluded.

Measures

The survey included two sets of questions. Those in the first set were designed to collect the demographic data, as explained below. The second set of questions asked about the participants’ CTS symptoms, for which purpose we used a validated Arabic version of the Boston carpal tunnel questionnaire (BCTQ-A). The original BCTQ developed by Levine et al. [19] has demonstrated excellent reliability and reproducibility and has been shown to be a valid instrument for screening the symptoms of CTS in previous studies [20-25]. The BCTQ-A used in this study has an intraclass correlation coefficient of 0.8 [21].

Demographic questions

The demographic questions on the survey asked about age and gender in order to determine the relationship, if any, between these characteristics and CTS symptoms. Thus, the women were asked whether they were pregnant to account for the increased prevalence of symptoms among pregnant women [26], and height and weight were obtained to calculate the BMI. We gathered additional data about social status, tobacco use, and exercise to assess the significance of protective and harmful factors and about hand dominance to assess whether right- or left-handedness is more associated with CTS symptoms. We also asked the participants whether they were currently working or retired, their level of education, the subjects that they taught, and whether they worked on-site or virtually to determine whether these factors were associated with CTS symptoms. The survey also collected information on the years that the participants had spent teaching and the amount of time that they had spent writing using a pen, keyboard, and/or blackboard to assess any association between these factors and CTS symptoms. Lastly, we asked whether the participants had been clinically diagnosed with CTS by a doctor to estimate their self-reporting of CTS and about other medical co-morbidities that could correlate with the symptoms of CTS.

Measurement of CTS symptoms

The participants responded to the BCTQ-A scale using a five-point Likert scale according to the severity of the participants’ symptoms, with 1 indicating no symptoms and 5 indicating the greatest severity. Table S1 provides further details about the responses as well as their coding.

We sought to compare teachers who had clinically confirmed CTS with clinically uncertain subjects as well as symptomatic with asymptomatic teachers. Symptomatic teachers were defined as those with at least one moderate to severe symptom in the hand and/or wrist identified on the BCTQ-A’s symptom severity and functional status scales (response codes 3 to 5 in).

Sample size calculation

Given that more than 100,000 teachers work in Riyadh, the estimated sample size was 385 participants at a 95% confidence interval (CI) [27]. Our initial sample included 763 potential participants. After excluding those who did not meet the eligibility criteria (i.e., were not teachers and did not work in Riyadh), our final sample size was 490 participants.

Statistical analysis

We used RStudio (R version 4.1.1) for the statistical analysis. The descriptive statistics included the frequencies and percentages for the categorical data and means and standard deviations (SDs) for the numerical data. We assessed the prevalence of CTS using a one-sample proportions test with continuity correction, presenting the prevalence along with the respective 95% CI. To conduct the univariate association analyses, we used a Wilcoxon rank sum test for the continuous variables and Fisher’s exact test or Pearson’s Chi-squared test for the categorical variables. We assessed the independent risk factors for CTS by constructing multivariate binary logistic regression models. The variables that showed significant association with the clinically confirmed diagnosis of CTS served as the independent variables in the first model. In the second model, we incorporated the factors that were significantly associated with having at least one moderate-to-severe symptom in the hands and/or wrists based on the BCTQ scale. We checked the assumptions of both models, and the results showed no significant multicollinearity, with the variance inflation factors being generally < 5. Additionally, the numerical variables were linearly associated with the dependent outcome variables in both models.

The results of the regression analysis are expressed using the OR and 95% CIs, with p < 0.05 indicating statistical significance.

Ethics approval and consent to participate

We obtained ethical approval for this study from King Abdullah International Medical Research Center, Ministry of National Guards Health Affairs, Riyadh, Saudi Arabia (protocol number NRC21R/539/12). The center’s IRB committee approved the informed consent form given to each participant before the study. Participation in this study was entirely voluntary, and the participants’ anonymity was secured in a manner consistent with the ethical considerations in the Declaration of Helsinki.

## Results

Sociodemographic and occupational characteristics

The present study includes data from the 490 teachers in the sample. Overall, 304 (62.0%) were women, most were married (84.1%), and most were right-handed (91.8%). There were significant gender differences between the male and female participants in terms of the proportions of those who were single (14.5% and 8.9%, respectively; p<0.0001), active smokers (21.0% and 1.0%; p<0.0001), exercised (48.4% and 33.2%; p<0.0001) and were right-handed (88.2% and 94.1%; p = 0.020, Table 1).

Concerning the participants’ specific duties, the most common subjects that they taught were math (17.8%), Arabic (15.3%), and religious education (9.8%). Table 1 also shows their active teaching, years of experience, and amount of time spent using a pen, keyboard, and/or blackboard. Significantly higher proportions of the male participants than the female participants were teaching onsite (90.3% and 75.0%, respectively; p<0.0001) and in a middle school (40.3% and 27.0%; p=0.001), while the female participants had spent on average more time using a pen, keyboard, and/or blackboard (3.5 ± 2.0 and 3.1 ± 1.7 hours daily; p = 0.028, Table 1).

**Table 1:** Sociodemographic and occupational characteristics *Data is based on 13 available records

Parameter	Category	Overall, N = 490	Male, N = 186	Female, N = 304	p
Age	Mean ± SD	40.8 ± 8.0	41.2 ± 8.6	40.6 ± 7.6	0.594
BMI	Underweight	4 (0.8%)	1 (0.5%)	3 (1.0%)	0.218
	Normal	134 (27.3%)	42 (22.6%)	92 (30.3%)	
	Overweight	207 (42.2%)	87 (46.8%)	120 (39.5%)	
	Obese	145 (29.6%)	56 (30.1%)	89 (29.3%)	
Marital status	Single	54 (11.0%)	27 (14.5%)	27 (8.9%)	<0.0001
	Married	412 (84.1%)	159 (85.5%)	253 (83.2%)	
	Divorced / widowed	24 (4.9%)	0 (0.0%)	24 (7.9%)	
Pregnant	Yes	NA	NA	15 (5.1%)	
Active smoker	Yes	42 (8.6%)	39 (21.0%)	3 (1.0%)	<0.0001
Exercise	Yes	191 (39.0%)	90 (48.4%)	101 (33.2%)	<0.0001
Hand dominance	Right-handed	450 (91.8%)	164 (88.2%)	286 (94.1%)	0.020
	Left-handed	40 (8.2%)	22 (11.8%)	18 (5.9%)	
Teach onsite or virtually	Onsite	396 (80.8%)	168 (90.3%)	228 (75.0%)	<0.0001
	Virtual	94 (19.2%)	18 (9.7%)	76 (25.0%)	
Which level do you teach	Kindergarten	15 (3.1%)	1 (0.5%)	14 (4.6%)	0.001
	Elementary school	136 (27.8%)	42 (22.6%)	94 (30.9%)	
	Middle school	157 (32.0%)	75 (40.3%)	82 (27.0%)	
	High school	182 (37.1%)	68 (36.6%)	114 (37.5%)	
Currently working or retired	Currently working	462 (94.3%)	178 (95.7%)	284 (93.4%)	0.292
	Retired	28 (5.7%)	8 (4.3%)	20 (6.6%)	
If retired, how long since you retired*	Mean ± SD	6.1 ± 3.8	3.0 ± 0.1	6.3 ± 3.9	0.500
How long have you been teaching	Mean ± SD	14.8 ± 8.2	15.3 ± 8.2	14.6 ± 8.3	0.316
Duration of using a pen/keyboard/board (hours)	Mean ± SD	3.4 ± 1.9	3.1 ± 1.7	3.5 ± 2.0	0.028

Medical history and characteristics of CTS

**Figure 1:**
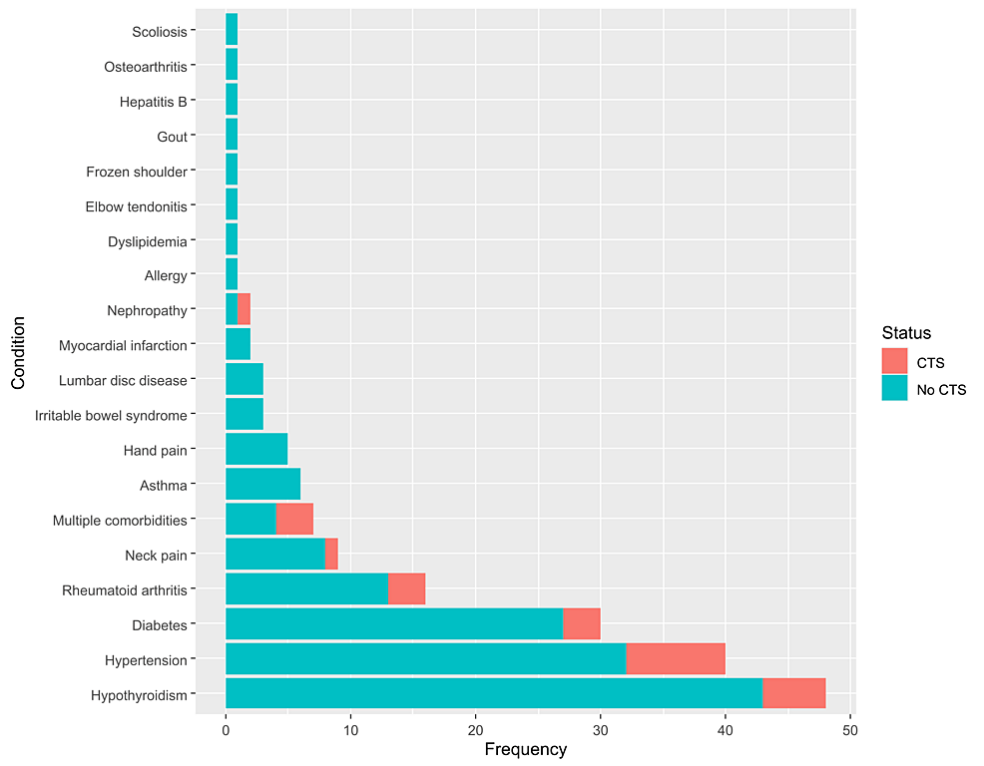
The distribution of chronic conditions among teachers.

One hundred and seventy-nine of the participants reported a positive history of a chronic condition (36.5%), most often hypothyroidism (26.8%), hypertension (22.3%), and diabetes (16.8%, Figure 1). Notably, 45 of the participants indicated that they had been diagnosed with CTS, for a prevalence of 9.2% (95% CI = 6.8 to 12.2). There was no statistically significant difference between the male and female participants in terms of the prevalence of CTS (10.2% and 8.6%, respectively; p = 0.536).

Factors associated with self-reported CTS

We found self-reported CTS to be significantly associated with being left-handed rather than right-handed (22.2% and 6.7%, respectively; p = 0.002) and retired rather than actively working (28.9% and 3.4%; p < 0.0001). Additionally, the teachers who self-reported CTS were significantly older than those who did not (44.4 ± 8.3 and 40.5 ± 7.9 years; p = 0.003), and they had been teaching for longer periods (17.7 ± 8.4 and 14.5 ± 8.2 years; p = 0.023). Furthermore, CTS was significantly associated with longer durations of using a pen, keyboard, and/or blackboard (4.0 ± 1.7 and 3.3 ± 1.9 hours per day; p = 0.003, Table 2). The multivariate analysis, then, indicated that the independent risk factors for CTS included left-hand dominance (OR = 4.10, 95% CI, 1.66 to 9.56, p = 0.001), being retired (OR = 9.67, 95% CI, 3.62 to 26.4, p < 0.0001), and having used a pen, keyboard, and/or blackboard for long periods of time (OR = 1.21, 95% CI, 1.03 to 1.42, p = 0.018; Table 3).

**Table 2:** Factors associated with self-reported CTS and having moderate to severe symptoms on BCTQ.

Parameter	Category	Self-reported CTS			Moderate to severe symptoms (BCTQ)			
		No, N = 445	Yes, N = 45	p	No, N = 294	Yes, N = 196	p	
Age	Mean ± SD	40.5 ± 7.9	44.4 ± 8.3	0.003	39.8 ± 7.7	42.4 ± 8.2	<0.0001	
Gender	Male	167 (37.5%)	19 (42.2%)	0.536	136 (46.3%)	50 (25.5%)	<0.0001	
	Female	278 (62.5%)	26 (57.8%)		158 (53.7%)	146 (74.5%)		
Pregnant	Yes	13 (4.8%)	2 (7.7%)	0.629	5 (3.3%)	10 (6.9%)	0.148	
BMI	Underweight	4 (0.9%)	0 (0.0%)	0.395	1 (0.3%)	3 (1.5%)	0.054	
	Normal	125 (28.1%)	9 (20.0%)		85 (28.9%)	49 (25.0%)		
	Overweight	189 (42.5%)	18 (40.0%)		132 (44.9%)	75 (38.3%)		
	Obese	127 (28.5%)	18 (40.0%)		76 (25.9%)	69 (35.2%)		
Marital status	Single	52 (11.7%)	2 (4.4%)	0.283	37 (12.6%)	17 (8.7%)	0.354	
	Married	372 (83.6%)	40 (88.9%)		244 (83.0%)	168 (85.7%)		
	Divorced / widowed	21 (4.7%)	3 (6.7%)		13 (4.4%)	11 (5.6%)		
Active smoker	Yes	39 (8.8%)	3 (6.7%)	0.786	31 (10.5%)	11 (5.6%)	0.056	
Exercise	Yes	169 (38.0%)	22 (48.9%)	0.153	118 (40.1%)	73 (37.2%)	0.520	
Hand dominance	Right-handed	415 (93.3%)	35 (77.8%)	0.002	276 (93.9%)	174 (88.8%)	0.043	
	Left-handed	30 (6.7%)	10 (22.2%)		18 (6.1%)	22 (11.2%)		
Teach onsite or virtually	Onsite	360 (80.9%)	36 (80.0%)	0.884	240 (81.6%)	156 (79.6%)	0.574	
	Virtual	85 (19.1%)	9 (20.0%)		54 (18.4%)	40 (20.4%)		
Which level do you teach	Kindergarten	14 (3.1%)	1 (2.2%)	0.438	6 (2.0%)	9 (4.6%)	0.404	
	Elementary school	126 (28.3%)	10 (22.2%)		82 (27.9%)	54 (27.6%)		
	Middle school	145 (32.6%)	12 (26.7%)		98 (33.3%)	59 (30.1%)		
	High school	160 (36.0%)	22 (48.9%)		108 (36.7%)	74 (37.8%)		
Currently working or retired	Currently working	430 (96.6%)	32 (71.1%)	<0.0001	288 (98.0%)	174 (88.8%)	<0.0001	
	Retired	15 (3.4%)	13 (28.9%)		6 (2.0%)	22 (11.2%)		
How long have you been teaching	Mean ± SD	14.5 ± 8.2	17.7 ± 8.4	0.023	14.3 ± 8.2	15.7 ± 8.2	0.048	
Duration of using a pen/keyboard/board (hours)	Mean ± SD	3.3 ± 1.9	4.0 ± 1.7	0.003	3.0 ± 1.6	3.9 ± 2.1	<0.0001	

Factors associated with moderate to severe symptoms on the BCTQ

The participants who reported having moderate to severe symptoms (i.e., with Likert scores from 3 to 5) for any of the 19 items on the BCTQ-A were considered to have positive symptoms. The teachers with moderate to severe symptoms were significantly older than those without such symptoms (42.4 ± 8.2 and 39.8 ± 7.7 years, respectively; p<0.0001), had taught for longer periods (14.3 ± 8.2 and 15.7 ± 8.2 years; p=0.048), and had used a pen, keyboard, and/or blackboard for longer periods (3.0 ± 1.6 and 3.9 ± 2.1 hours per day; p<0.0001). These symptoms were also significantly associated with the female gender (74.5% and 53.7%; p<0.0001), left-handedness (11.2% and 6.1%, p = 0.043), and being retired (11.2% and 2.0%; p<0.0001; Table 2). The multivariate analysis showed that experiencing moderate to severe symptoms was independently associated with the female gender (OR = 2.62, 95% CI, 1.72 to 4.06, p < 0.0001), being retired (OR = 3.62, 95% CI, 1.41 to 10.6, p = 0.011), and left-hand dominance (OR = 2.23, 95% CI, 1.09 to 4.61, p = 0.018). Additionally, older teachers (OR = 1.04, 95% CI, 1.00 to 1.09, p = 0.034) and those who had been using a pen, keyboard, and/or blackboard for relatively longer times (OR = 1.30, 95% CI, 1.17 to 1.46, p < 0.0001) were more likely to report moderate to severe symptoms (Table 3).

**Table 3:** Multivariate regression analysis for the risk factors of self-reported CTS and symptomatic pain based on the BCTQ. NS: non-significant in the univariate analysis; Ref: reference category

Parameter	Category	Self-reported CTS			Moderate to severe symptoms (BCTQ)		
		OR	95% CI	p-value	OR	95% CI	p-value
Gender	Male	NS	NS	NS	Ref	Ref	Ref
	Female	NS	NS	NS	2.62	1.72, 4.06	<0.0001
Age	Numeric	1.00	0.93, 1.06	0.886	1.04	1.00, 1.09	0.034
Hand dominance	Right-handed	Ref	Ref	Ref	Ref	Ref	Ref
	Left-handed	4.10	1.66, 9.56	0.001	2.23	1.09, 4.61	0.028
Currently working or retired	Currently working	Ref	Ref	Ref	Ref	Ref	Ref
	Retired	9.67	3.62, 26.4	<0.0001	3.62	1.41, 10.6	0.011
How long have you been teaching	Numeric	1.04	0.98, 1.10	0.185	0.99	0.96, 1.03	0.712
Duration of using a pen/keyboard/board	Numeric	1.21	1.03, 1.42	0.018	1.30	1.17, 1.46	<0.0001

## Discussion

Our analysis showed that teachers who were female, older, left-handed, retired, and had spent long hours using a pen, keyboard, and/or blackboard were more likely to self-report CTS and to exhibit moderate to severe symptoms. The time spent teaching may also play a role in developing CTS, for teachers who had spent relatively long periods teaching experienced the symptoms of CTS more than those who spent less.

The participants’ responses to the BCTQ-A indicated that the prevalence of moderate to severe symptoms was 40.0%. We found the prevalence of self-reported CTS among school teachers to be 9.1%, which is, surprisingly, higher than that reported in the general population [1,28]. Moreover, previous researchers estimated the percentage of wrist and/or hand symptoms in Saudi teachers from 7.4% to 22.1% [13-16]. Younis et al. [18] found that 62% of the symptomatic school teachers whom they studied had clinically confirmed CTS, but this relatively high percentage may be attributable to the fact that they included only symptomatic teachers in their sample. Despite the large teaching workforce in Saudi Arabia, there is a gap in the literature regarding the occurrence of MSDs among the country’s educators. Thus, whereas the prevalence of hand and/or wrist symptoms reported in previous studies was lower than the prevalence of back and neck pain, we found that 40.0% of the teachers in our sample (196 of 490) reported moderate to severe symptoms that could negatively impact their work productivity.

Gender was significantly associated with the prevalence of CTS symptoms in the current study, with female teachers being more affected by symptoms than male teachers. Likewise, Younis et al. [18], Atroshi et al. [1] and De Krom et al. [28] all found the prevalence of CTS to be higher among women than men in the general population. So also, Erick et al. [3], in a systematic review, found that women were more prone to severe wrist/hand pain than men. A possible explanation for this gender difference is that females generally have smaller wrists than men and/or a lower pain threshold for reporting these symptoms and/or that men may hesitate to report such symptoms out of embarrassment [29].

We also found the number of hours that teachers spent writing to be associated with the prevalence of CTS symptoms. Thus, the teachers with symptoms had spent an average of 3.9 ± 2.1 hours per day using a pen, keyboard, and/or blackboard. Similarly, Younis et al. [18] reported the average working time for teachers with CTS in the study to be 6.8 hours per day. Additionally, Cardoso et al. demonstrated that the prevalence of MSDs increased with the amount of time that the teachers in their study spent teaching [30]. Erick et al. showed further that both the time spent by teachers on teaching and awkward positioning of the arm while working can contribute significantly to the development of MSDs, and that teachers who work with their arms in awkward positions were 1.59 times more likely to develop wrist and/or hand pain than those who did not [31]. Other researchers have likewise hypothesized that an occupational causative factor of CTS among teachers is the position of the hand and head while writing on blackboards [32]. The odds ratio of 1.30 for writing using a pen, keyboard, and/or blackboard that we found, however, indicates that the association was not significant. Accordingly, not only the time that teachers spend teaching and writing but also the way in which they perform their tasks may be indicators of the development of CTS.

More than a third (36.5%) of the teachers in the current study had had previous medical problems. Of these comorbidities, diabetes, hypothyroidism, and a relatively high BMI are the most commonly known risk factors of CTS [33-35]. Another study linked metabolic disorders such as diabetes specifically to CTS [36]. Therefore, such risk factors, which have become widespread in populations worldwide in recent years, may be contributing to the status of CTS as an emerging workplace health issue.

Limitations

The limitations of this study include, in the first place, the design. Specifically, since we conducted a cross-sectional study, the results may not be generalizable across Saudi Arabia let alone to other populations. Furthermore, Because the study was survey-based, it was subject to recall bias and subjective differences in the reporting of the items in the BCTQ-A, particularly those related to pain. Another limitation is the convenience sampling method that we employed: because of the COVID-19 pandemic, we could only reach the intended population through social media platforms, thus introducing a possible source of sampling bias. The use of the BCTQ, a validated tool for reporting CTS symptoms, increased the rigor of this study, but reinforcing these findings with a clinical approach would have provided more precise and valuable results. Thus, future research along these lines could include a clinical dimension and broader regional coverage and make use of a larger sample size and a different design, such as prospective studies.

## Conclusions

We found the prevalence of CTS symptoms to be 40.0% among the teachers working in Riyadh whom we surveyed. This proportion is greater than that reported in the general population. Further assessment of the risk factors discussed here is needed. Additionally, we recommend that teachers consult medical advice when they suspect that they are developing CTS symptoms since early diagnosis and treatment are associated with favorable outcomes. There is a particular need for such research given the increase in the general population of such CTS risk factors as diabetes, hypothyroidism, and high BMI.
